# Esketamine Reduces Lung Injury Caused by Limb Ischemia-Reperfusion by Regulating Oxidative Stress *via * the TLR4/NF-κB/NLRP3 Pathway

**DOI:** 10.2174/0118715303393744250423100211

**Published:** 2025-04-29

**Authors:** Meng Wang, Qian Ma, Wenjuan Wang, Jiawei Cun, Heng Wen

**Affiliations:** 1 Department of Anesthesiology, General Hospital of Ningxia Medical University, Yinchuan, 750004, China;; 2 Ningxia Medical University, Yinchuan, 750004, China;; 3 Department of Anesthesiology, The First Affiliated Hospital of Zhejiang University School of Medicine, Hangzhou, 310006, China

**Keywords:** Esketamine, acute lung injury, limb ischemia-reperfusion, TLR4/NF-κB/NLRP3 pathway, inflammatory cytokine, oxidative stress

## Abstract

**Background:**

Esketamine has shown promise in mitigating tissue damage caused by ischemia-reperfusion injury, making it a potential therapeutic candidate for acute lung injury (ALI) induced by limb ischemia-reperfusion (LIR-ALI).

**Objective:**

This study sought to explore the role and mechanism of esketamine in the LIR-ALI rat model.

**Methods:**

The effects of esketamine on the LIR-ALI rats model were evaluated through histopathological examination, assessment of pulmonary edema, measurement of MDA and SOD levels, and analysis of inflammatory cytokine levels (IL-1β, *etc*.) in the bronchoalveolar fluid (BALF) and serum. Western blot analysis was used to assess the expressions of TLR4, NF-κB, and NLRP3. TLR4 agonist, LPS, was used to validate the role of NF-κB/NLRP3 pathway in LIR-ALI.

**Results:**

Esketamine significantly alleviated LIR-induced ALI by reducing pulmonary edema, inflammatory cell infiltration, and oxidative stress. Elevated MDA content and suppressed SOD activity were significantly reversed by esketamine, along with inactivity of the TLR4/NF-κB/NLRP3 pathway. Esketamine treatment reduced inflammatory response in BALF and serum. TLR4 activation by LPS reversed the ameliorative effects of esketamine on LIR-ALI.

**Conclusion:**

Esketamine protected against LIR-induced ALI by mitigating oxidative stress and suppressing the TLR4/NF-κB/NLRP3 axis. These findings highlight the potential therapeutic value of esketamine for ALI.

## 1 INTRODUCTION

LIR injury is recognized as a critical pathophysiological process often encountered in diverse clinical contexts, such as trauma emergencies and various surgical interventions [1]. This injury significantly compromises patient outcomes, potentially impacting prognosis and quality of life [1]. LIR injury precipitates damage in distant organs, with ALI being a significant manifestation, driven by complex pathophysiological mechanisms involving inflammatory mediators and oxidative stress [2], and its core mechanisms involve complex cascade reactions, prominently featuring oxidative stress and the activation of inflammatory mediators [3]. Therefore, in-depth research on LIR and the associated ALI is crucial in current basic and clinical research.

Esketamine, as the dextrorotatory enantiomer of ketamine, is a non-competitive N-methyl-D-aspartate (NMDA) receptor channel inhibitor. Compared to ketamine, esketamine exhibits higher pharmacological activity, lower effective dose requirements, and better respiratory and circulatory system stability, thus offering significant advantages in clinical applications [4, 5]. Current studies demonstrate that esketamine can exert anti-inflammatory and anti-oxidative effects *via* multiple mechanisms. Under mechanical ventilation, the application of esketamine can significantly reduce inflammatory response in lung tissue, and it upregulates the expression of IL-10 [6]. In addition, esketamine has also been shown to reduce inflammation-related tissue damage by inhibiting microglia-mediated inflammatory responses [7]. These findings strongly suggest the potential therapeutic value of esketamine in regulating cytokine balance and alleviating inflammatory responses.

In the LIR-induced inflammatory cascade, overactivation of NLRP3 can induce the release of pro-inflammatory cytokines and promote pyroptosis, thus amplifying inflammatory responses and tissue damage [8, 9]. Targeting the NLRP3 inflammasome represents a promising therapeutic strategy to mitigate inflammatory damage, preserve organ function, and improve patient outcomes [10]. In addition, LIR can also lead to oxidative stress, one of the consequences of which is an increase in the level of malondialdehyde (MDA). Elevated levels of MDA, indicative of heightened oxidative stress, are linked to the intensification of pulmonary inflammation and compromised lung function. This condition contributes to the development of pulmonary edema and impedes efficient gas exchange, as supported by recent findings [11, 12]. Therefore, in-depth exploration of the molecular mechanisms of LIR injury, especially the interaction between inflammatory cascade and oxidative stress-related pathways, is of significant academic and clinical translational importance.

Therefore, in this study, we sought to investigate the protective role of esketamine in LIR-induced ALI. Specifically, we investigated the role of esketamine in modulating oxidative stress and the TLR4/NF-κB/NLRP3 pathway in LIR-induced ALI rats. The results have elucidated its therapeutic implications for mitigating ALI under LIR-associated pathologies.

## 2 METHODS

### 2.1 Experimental Protocol

Thirty specific pathogen-free (SPF) grade female Sprague-Dawley (SD) rats, aged 8 weeks and weighing 180–220 g, were purchased from Guangdong Medical Laboratory Animal Center, China. Rats were housed under controlled environmental conditions, including a temperature of 22 ± 2°C, relative humidity of 50%–60%, and a 12-hour light/dark cycle. Standard laboratory chow and water were provided ad libitum. Rats were randomly assigned to one of the five groups (n = 6), including the sham group, ALI group, low-dose esketamine group (ESK-L), high-dose esketamine group (ESK-H), and LPS-induced group (ESK+LPS). In the sham group, the rats were intraperitoneally injected with normal saline. The ALI group comprised the LIR-ALI model rats, in which normal saline was intraperitoneally injected 5 minutes before reperfusion. In the ESK-L group, esketamine (230619BL, Jiangsu Hengrui Pharmaceutical Co., Ltd., China) was intraperitoneally injected at a dose of 0.8 mg/kg 5 minutes before reperfusion. In the ESK-H group, esketamine was intraperitoneally injected at a dose of 1.6 mg/kg 5 minutes before reperfusion. In the ESK+LPS group, lipopolysaccharide (LPS) (Solarbio, L8880) was administered intravenously, and esketamine was intraperitoneally injected simultaneously at a dose of 1.6 mg/kg 5 minutes before reperfusion.

To ensure clinical relevance, inter-species differences were considered during the selection of these dosages. Specifically, acknowledging the pharmacokinetic and pharmacodynamic variations between rats and humans, the esketamine doses used in this study (0.8 mg/kg and 1.6 mg/kg [13]) were derived from clinically administered doses (0.25 mg/kg and 0.5 mg/kg) using a recommended conversion factor of 3.2 [14]. This conversion was performed to simulate therapeutic scenarios observed in clinical practice. All animal experiments were approved by the ethics committee of the General Hospital of Ningxia Medical University (KYLL-2022-0531).

### 2.2 LIR-ALI Model

After a 12-hour fast with free access to water, rats were anesthetized using pentobarbital sodium (40 mg/kg, i.p., P3761, Sigma). Bilateral inguinal incisions were made to expose the femoral arteries. These arteries were then occluded using vascular clamps. Concurrently, tourniquets were applied at the root of each thigh to induce limb ischemia for 2 hours. Five minutes before reperfusion, designated groups received esketamine intraperitoneally at either a low or high dose, as described in the experimental protocol. Subsequently, the vascular clamps and tourniquets were released to initiate reperfusion. Following the initiation of reperfusion, the inguinal incisions were closed with sutures. As the rats began to recover from anesthesia after wound closure, they received a single subcutaneous injection of carprofen (5 mg/kg) for postoperative analgesia. Reperfusion was maintained for 12 hours. At the 12-hour time point, peripheral blood was collected from living rats *via* the tail vein. Immediately following blood collection, rats were euthanized with an overdose of pentobarbital sodium (100 mg/kg, i.p., P3761, Sigma). Post-euthanasia, bronchoalveolar lavage fluid (BALF) and lung tissues were harvested. In the sham group, all surgical procedures were performed identically, except for the application of vascular clamps and tourniquets. Sham animals also received carprofen post-suturing. Pain and distress were minimized with optimal conditions and anesthetics.

### 2.3 H&E Staining

Lung tissue samples from each group of rats were fixed in a 4% paraformaldehyde solution. Afterward, fixed tissues were subjected to dehydration, clearing, and paraffin embedding before sectioning. The sections were stained with hematoxylin, rinsed with distilled water, and then blued in tap water. Subsequently, eosin was applied for counterstaining, which was followed by another rinse with distilled water. The sections were then dehydrated using a graded ethanol series (95%-100%). After dehydration, the sections were cleared in xylene for 10 minutes and finally mounted with neutral resin. Following mounting, lung tissue morphology was observed under a light microscope (×200), and images were documented. A H&E staining kit (G1076, ServiceBio) was used for staining [15, 16].

### 2.4 Pulmonary Edema Evaluation

Pulmonary edema severity was evaluated *via* the wet/dry weight (W/D) ratio method. The previous methodology was followed [17]. Briefly, lung tissue surfaces were gently blotted dry, and wet weight (W) was measured. Samples were then dried at 80^o^C for 24 hours. Finally, the W/D ratio and total lung water content (TLW%) were calculated as follows:

Lung W/D weight ratio (W/D):


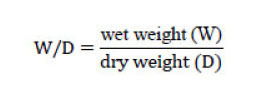


Total lung water (TLW%):


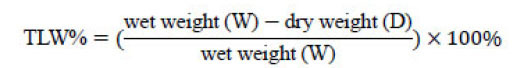


### 2.5 Determination of MDA Content and Superoxide Dismutase (SOD) Activity in Lung Tissue

Lung tissue was collected from rats and homogenized and the supernatant was collected after centrifugation. Commercial assay kits were employed to determine the MDA and SOD contents in the lung tissues of each group. Malondialdehyde was measured based on the absorbance of the product formed by its reaction with thiobarbituric acid at 532 nm using a microplate reader (Thermo Fisher Scientific, USA). A malondialdehyde (MDA) content assay kit (Solarbio, BC0025) was used, with its application performed in accordance with a previous study [18]. SOD activity was evaluated based on its enzymatic reaction, with absorbance recorded at 450 nm using the microplate reader. A superoxide dismutase (SOD) activity assay kit (Solarbio, BC5156) was used according to the previously reported method [19, 20].

### 2.6 ELISA

The levels of pro-inflammatory cytokines in serum and BALF were quantified using ELISA kits, including rat IL-1β ELISA kit (Solarbio, SEKR-0002), rat IL-6 ELISA kit (Solarbio, SEKR-0005), and rat TNF-α ELISA kit (Solarbio, SEKR-0009), according to the manufacturers’ instructions. Briefly, supernatants from serum, BALF, and lung homogenates were collected. Subsequently, OD450 nm of each sample was detected and recorded using a microplate reader (Thermo Fisher Scientific, USA). Based on the standard curve, the concentrations of IL-1β, IL-6, and TNF-α in the samples were calculated [21].

### 2.7 Western Blot Assay

The total protein was isolated from tissues, and protein concentration was determined using the BCA method. Equal amounts of protein were separated by SDS-PAGE and transferred onto PVDF membranes using a wet transfer method. Membranes were blocked with 5% non-fat dry milk in tris-buffered saline containing 0.1% Tween 20 (TBST) for 1 hour at 25°C. After three washes with TBST (10 min each), membranes intended for target protein detection were incubated overnight at 4°C with primary antibodies diluted in TBST against TLR4 (19811-1-AP, 1:1,000, Proteintech, Chicago, USA), NF-κB p65 (#8242, 1:1,000, Cell Signaling Technology, CST), and NLRP3 (ab263899, 1:1,000, Abcam, Cambridge, UK). For normalization, GAPDH (#5174, 1:1,000; CST) was detected on separate membranes prepared and processed in parallel using identical sample loadings and incubation conditions. Following primary antibody incubation, all membranes were washed three times with TBST (10 min each) and then incubated with HRP-conjugated goat anti-rabbit secondary antibody (diluted 1:5000 in TBST) for 1 hour at room temperature on a shaker. After three final washes with TBST (10 min each), protein bands were visualized using an ECL reagent and captured with a Multi- function Imaging System (Bio-Rad, USA). The integrated optical density (IOD) of each band was quantified using ImageJ software. Relative protein levels were calculated by normalizing the IOD of the target protein to the IOD of GAPDH from the corresponding sample run on the parallel blot [22].

### 2.8 TUNEL Assay

Following deparaffinization in xylene and dehydration, tissue sections were washed with PBS (pH 7.4) and digested with 20 μg/mL proteinase K at 37°C for 20 minutes to expose DNA fragments. Endogenous peroxidase activity was blocked, and then incubation was performed with a TUNEL reaction mixture (containing TdT enzyme and fluorescein-labeled dUTP) at 37^o^C for 60 minutes in a dark humidified chamber, followed by incubation with HRP-conjugated reagent and DAB substrate under light-protected conditions. Then nuclei were counterstained with hematoxylin. TUNEL-positive apoptotic cells (brown-stained nuclei) were visualized under a light microscope (×200 magnification). TUNEL assay kit was provided by Beyotime (Shanghai, China) [23, 24].

### 2.9 Statistical Analysis

All data have been presented as mean ± standard deviation (SD). Data analysis was performed using GraphPad Prism 8 software. In this study, data were analyzed using ANOVA, followed by Tukey’s post-hoc test. A *p*-value of less than 0.05 (*p* < 0.05) was considered statistically significant. All experiments were repeated at least three times.

## 3 RESULTS

### 3.1 Effects of Esketamine on Pathology and Pulmonary Edema in the LIR-ALI Rat Model

**Fig. (1):**
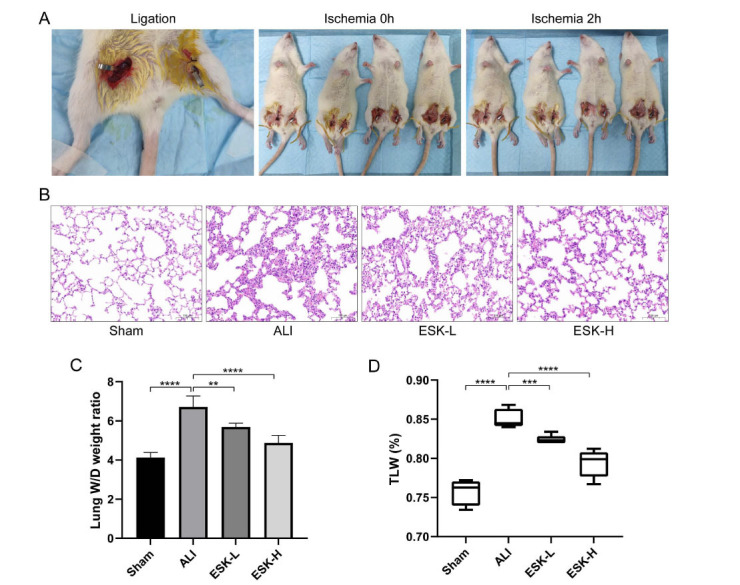
Effects of esketamine on pathology and pulmonary edema in the LIR-ALI rat model. (**A**) Establishment of the LIR-ALI model. Limb ischemia was induced by clamping the femoral artery and applying a tourniquet for 2 hours, followed by 2 hours of reperfusion by releasing the clamp and tourniquet. (**B**) H&E staining of lung tissue pathology in each group (×200). The arrows represent alveolar septal thickness. (**C**) Analysis of the lung W/D ratio. (**D**) Analysis of TLW %. *p<0.05, **p<0.01, ***p<0.001, ****p<0.0001.

Pathological examination of lung tissue was performed in the LIR-ALI rat model (Fig. **1A**). H&E staining revealed extensive inflammatory cell infiltration in the lung tissues of LIR-ALI rats, along with changes in alveolar septal thickness. Following treatment with low-dose and high-dose ESK, the lung tissue damage showed significant improvement, with the high-dose group demonstrating particularly notable intervention effects (Fig. **1B**). Subsequent analysis of lung tissue showed the W/D and TLW content in LIR-ALI rats to be significantly increased compared to the sham group. However, ESK treatment significantly rescued both the W/D and TLW content in the lung tissues of LIR-ALI rats (Figs. **1C** and **1D**).

### 3.2 Alleviation of Oxidative Stress and Tissue Apoptosis by ESK in LIR-ALI Rat Model

**Fig. (2):**
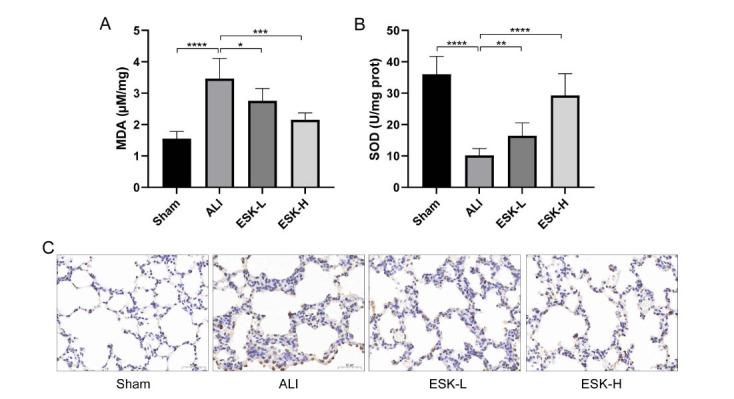
Alleviation of oxidative stress and apoptosis in lung tissue of the LIR-ALI rat model by ESK. (**A, B**) Detection of MDA and SOD levels in lung tissue using assay kits. (**C**) TUNEL assay for detecting apoptosis in rat lung tissue (magnification ×200). *p<0.05, **p<0.01, ***p<0.001, ****p<0.0001. The arrows represent cells positive for TUNEL staining.

To investigate the effect of esketamine, the SOD and MDA levels in lung tissue were measured. As shown in Fig. (**2**), the MDA content was significantly increased in LIR-ALI rats (Fig. **2A**), which was accompanied by a significant downregulation of SOD levels (Fig. **2B**) and a marked increase in apoptotic signals in the tissue (Fig. **2C**). However, following treatment with low-dose ESK (ESK-L) and high- dose ESK (ESK-H), the MDA levels in lung tissue were significantly reduced, while SOD levels were significantly upregulated, while TUNEL signals in the lung tissue were decreased (Figs. **2A-C**).

### 3.3 Effects of ESK on the TLR4/NF-κB/NLRP3 Pathway in Lung Tissue of the LIR-ALI Rat Model

**Fig. (3):**
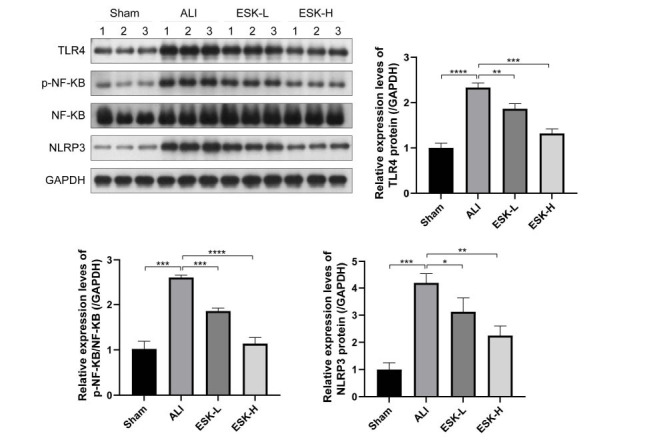
Effects of ESK on the TLR4/NF-κB/NLRP3 pathway in lung tissue of the LIR-ALI rat model. Western blot analysis was used to quantify the levels of TLR4, NF-κB, and NLRP3 in rat lung tissue. *p<0.05, **p<0.01, ***p<0.001, ****p<0.0001.

**Fig. (4):**
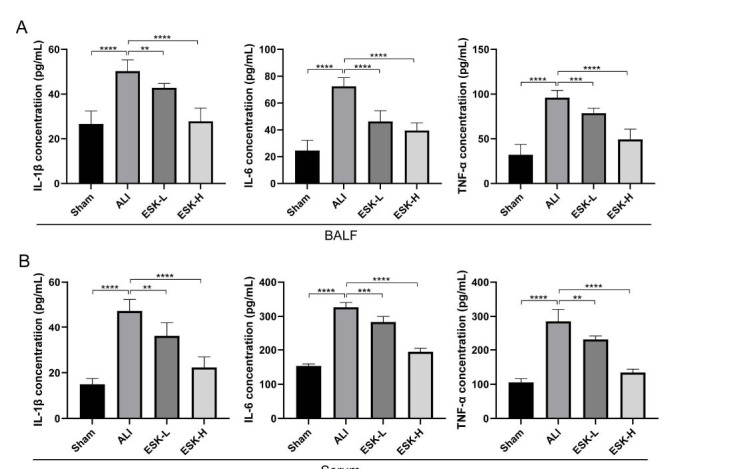
Effects of ESK on IL-1β, IL-6, and TNF-α in BALF and serum of the LIR-ALI rat model. (**A**) ELISA detection of IL-1β, IL-6, and TNF-α levels in BALF of each group. (**B**) ELISA detection of IL-1β, IL-6, and TNF-α levels in lung tissue of each group. *p<0.05, **p<0.01, ***p<0.001, ****p<0.0001.

To investigate the effect of esketamine intervention on inflammatory pathways, TLR4/NF-κB/NLRP3 pathway activation was evaluated using Western blot analysis. The levels of TLR4, NLRP3, and NF-κB were significantly upregulated in LIR-ALI rats (Figs. **3A-D**). However, after treatment with different doses of ESK, the expression levels of TLR4, NLRP3, and NF-κB in lung tissue showed a downward trend, with high-dose ESK exhibiting a stronger inhibitory effect on TLR4/NF-κB/NLRP3 pathway (Fig. **3**). The levels of IL-1β, IL-6, and TNF-α in BALF and serum significantly reversed in LIR-ALI rats after treatment with different concentrations of ESK (Figs. **4A** and **4B**).

### 3.4 TLR4 Activation Reversed the Protective Effects of ESK in the LIR-ALI Model

**Fig. (5):**
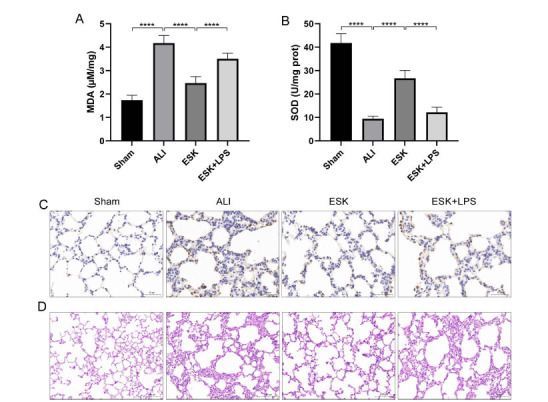
LPS-induced activation of TLR4 reversed the protective effects of ESK in the LIR-ALI model. (**A, B**) Detection of MDA content (**A**) and SOD activity (**B**) in lung tissue using assay kits. (**C**) TUNEL staining assay for detecting apoptosis in lung tissue. The arrows represent cells positive for TUNEL staining. (**D**) H&E staining of lung tissue pathology in each group (magnification ×200). *p<0.05, **p<0.01, ***p<0.001, ****p<0.0001. The arrows represent alveolar septal thickness.

To further confirm that ESK exerted protective effects in the LIR-ALI model by intervening in the TLR4/NF-κB/NLRP3 pathway, animals treated with ESK were subsequently treated with a TLR4 agonist. The MDA content and SOD activity in lung tissue were measured using assay kits. As shown in Fig. (**5**), the MDA content in the lung tissue of the LIR-ALI model was significantly increased, which was accompanied by a sharp decrease in SOD activity and a marked increase in TUNEL-positive cells (Figs. **5A-C**). ESK treatment significantly reversed the MDA content, SOD activity, and apoptosis in lung tissue (Figs. **5A-C**). However, this reversal effect was counteracted after treatment with the TLR4 agonist LPS, which diminished the protective effects of ESK on lung tissue (Figs. **5A-C**). H&E staining showed the protective effects of ESK on lung tissue to be negated following further treatment with LPS (Fig. **5D**).

**Fig. (6):**
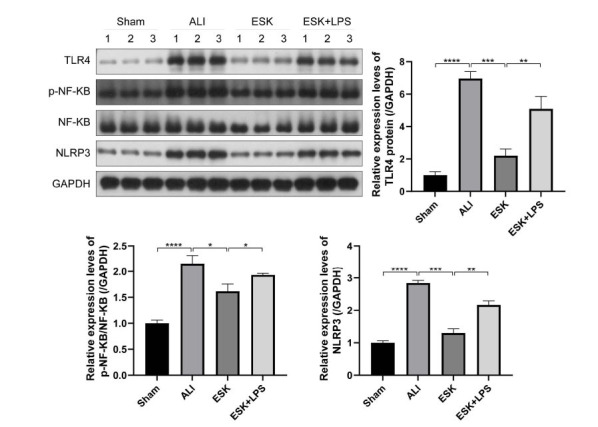
LPS-induced activation of TLR4 reversed the inhibitory effects of ESK on the TLR4/NF-κB/NLRP3 pathway in the LIR-ALI model. Western blot analysis was used to detect the expression levels of TLR4, p-NF-κB, NF-κB, and NLRP3 in lung tissue across different groups. *p<0.05, **p<0.01, ***p<0.001, ****p<0.0001.

**Fig.(7):**
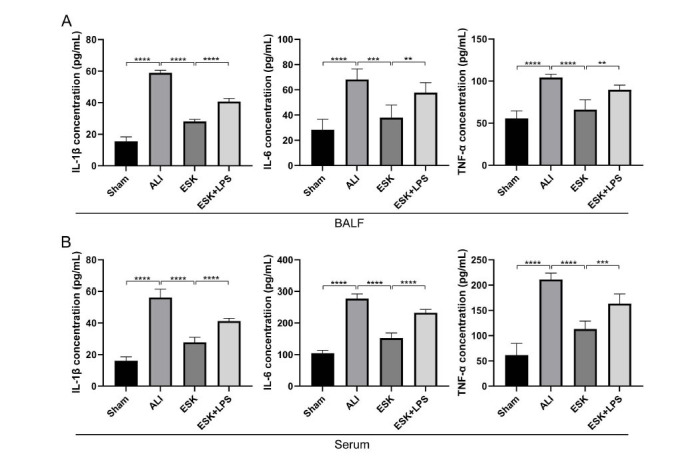
LPS-induced activation of TLR4 reversed the downregulation of inflammatory factors by ESK in the LIR-ALI model. ELISA detection of IL-1β, IL-6, and TNF-α levels in BALF (**A**) and lung tissues (**B**). *p<0.05, **p<0.01, ***p<0.001, ****p<0.0001.

Western blot analysis was employed to detect the suppressed expression of TLR4, NF-κB, and NLRP3 in the LIR-ALI model after treatment with esketamine. Results showed the effect of esketamine to be reversed by the TLR4 agonist LPS. This was evidenced by the upregulation of TLR4 and NLRP3 expression levels and increased phosphorylation of NF-κB (Fig. **6**). Further, ELISA experiments demonstrated the inhibitory effects of ESK on IL-1β, IL-6, and TNF-α levels in BALF and serum of the LIR-ALI model to be counteracted after LPS treatment, as these inflammatory factors were further upregulated. This suggests that LPS activation exacerbated the inflammatory response and diminished the protective efficacy of esketamine (Fig. **7A** and **7B**).

## 4 DISCUSSION

LIR is a common pathological process, frequently observed in hemorrhagic shock and natural disasters [25]. The occurrence of IR injury is mainly due to excessive free radicals attacking tissue cells that have regained blood supply, which is accompanied by intracellular calcium overload and tissue damage caused by the combined action of leukocytes [26]. In the lungs, LIR injury is prone to occur due to the high dependence on blood supply. LIR produces a large number of inflammatory mediators and oxygen-free radicals, which damage pulmonary microvessels and lead to cell damage [9]. Among them, pyroptosis is an important mechanism for the progression of LIR injury and is considered the most common form of inflammatory cell death [27]. Recent evidence confirms that LIR triggers NLRP3 inflammasome activation, initiating pyroptosis. This process significantly amplifies the inflammatory cascade and contributes to subsequent lung tissue damage [28, 29]. ALI is a significant complication of LIR, and its main characteristic is an excessive inflammatory response [26, 30]. In this process, the massive accumulation of neutrophils and the rapid release of pro-inflammatory factors [31] not only damage the pulmonary vascular endothelial and epithelial cells, but also increase vascular permeability, leading to pulmonary edema [30]. The W/D and TLW ratio of lung tissue can effectively reflect the degree of pulmonary edema and are important indicators for measuring lung injury [32]. Increased W/D and TLW ratios indicate increased pulmonary tissue capillary permeability and expansion of the capillary membrane space. The ALI group exhibited marked pathological alterations, including elevated W/D and TLW, indicative of aggravated pulmonary edema. Concurrently, diminished SOD activity, heightened levels of IL-1β, IL-6, and TNF-α, and upregulated expression of TLR4/NF-κB/NLRP3 pathway proteins collectively demonstrated exacerbated oxidative stress and inflammatory activation. These findings confirm the pivotal role of TLR4/NF-κB/NLRP3 signaling in mediating LIR-induced ALI.

Research suggests that inhibiting inflammation can reduce organ damage from LIR [33]. It also reduces inflammatory stress during lung surgery. Combined medication with esketamine can relieve pain, promote recovery, and reduce complications, offering a valuable approach to postoperative care [34]. It is pertinent to note that esketamine functions as an NMDA receptor antagonist, and currently, research has not yet determined the optimal dose for preventing ischemia-reperfusion injury. Insights from existing literature on a similar NMDA receptor antagonist, ketamine, suggest that both neuroprotective and neurotoxic effects are closely tied to dosage and administration frequency. Specifically, higher frequencies and larger doses of ketamine might lead to a significant reduction in glutamatergic synapse numbers due to increased long-term neurotoxicity [35]. Conversely, low-frequency (*e.g.*, 1-3 times weekly) or low-dose (*e.g.*, 10 mg/kg) ketamine administration has demonstrated significant neuroprotective benefits, such as increasing dendritic spine density, enhancing synaptic efficacy, and improving synaptic plasticity, without elevating synaptic glutamate levels or causing apparent neurotoxicity, thus maintaining neuroprotective and antidepressant effects [36]. While optimizing the dosage regimen requires further investigation, the findings of this study demonstrate esketamine's potential in the context of LIR. Compared to the ALI group, the ESK group showed significant protection. Esketamine downregulates pro-inflammatory factors, like IL-1β, IL-6, and TNF-α. It also significantly inhibits TLR4, NF-κB, and NLRP3 signaling. Thus, esketamine reduces inflammatory damage *via* suppressing TLR4/NF-κB/NLRP3 signaling pathway. Additionally, the ESK group had increased SOD activity and decreased MDA concentration, alleviating oxidative stress. These results confirm esketamine's protective effect against LIR-induced ALI. LPS activates TLR4 signaling, which subsequently promotes the production and release of pro-inflammatory cytokines, contributing to the exacerbation of lung inflammation and injury [37]. In this experiment, the ESK+LPS group showed that LPS further aggravated lung inflammation and oxidative stress. This was evident through increased IL-1β, IL-6, and TNF-α expressions, enhanced TLR4, NF-κB, and NLRP3 signaling, decreased SOD activity, and increased MDA concentration. However, even with LPS-induced inflammation, esketamine still alleviated oxidative stress and inflammatory response, and inhibited TLR4, NF-κB, and NLRP3 signaling. This highlights its potential to protect against infection-induced inflammation.

While this study provides insights into the protective effects of esketamine against LIR-induced ALI *via* the TLR4/NF-κB/NLRP3 pathway, certain limitations should be acknowledged, which can pave the way for future research. Firstly, the validation of the proposed mechanism, although supported by our findings, remains relatively preliminary. Future studies could employ more specific molecular techniques, such as using pathway inhibitors or activators in conjunction with esketamine, or *in vitro* experiments, to further elucidate the precise interactions and downstream effects, including a more detailed analysis of pyroptosis markers beyond NLRP3 activation. Secondly, this study did not directly assess potential systemic side effects or other detailed systemic safety indicators within this specific LIR model. Addressing these potential risks and defining a safe therapeutic window are crucial translational challenges that warrant investigation in future preclinical studies, potentially including dose-response analyses and behavioral assessments. Thirdly, our experimental design utilized a single intervention time point for esketamine administration. Consequently, the critical question of whether the timing of esketamine treatment significantly influences its protective efficacy against LIR injury remains unanswered. Future research should explore various administration schedules to determine the optimal therapeutic window for maximizing esketamine's benefits in this context. Addressing these limitations can deepen our understanding of esketamine's role in LIR-induced ALI and facilitate its potential clinical translation.

## CONCLUSION

This study has successfully established a rat model of LIR-induced ALI, characterized by significant pulmonary edema, heightened oxidative stress, and robust inflammatory responses. Our findings have confirmed the critical involvement of the TLR4/NF-κB/NLRP3 signaling pathway in the pathogenesis of LIR-induced ALI, as evidenced by the upregulation of its key protein components in the ALI group. The central finding of this research work was the demonstration of esketamine's significant protective effects against LIR-induced ALI. The administration of ESK effectively mitigated pulmonary edema, attenuated oxidative stress by enhancing SOD activity and reducing MDA concentration, and suppressed the inflammatory cascade by downregulating the levels of pro-inflammatory cytokines IL-1β, IL-6, and TNF-α. Crucially, ESK treatment led to significant inhibition of the TLR4/NF-κB/NLRP3 signaling pathway activation. Furthermore, even when challenged with LPS to exacerbate inflammation, ESK retained its capacity to alleviate oxidative stress and dampen the inflammatory response, including the suppression of TLR4/NF-κB/NLRP3 signaling, highlighting its potential efficacy even in complex inflammatory scenarios.

Collectively, these results strongly suggest that esketamine confers protection against LIR-induced ALI, at least in part, by modulating oxidative stress and inhibiting the TLR4/NF-κB/NLRP3 inflammatory pathway. While acknowledging the study's limitations, including the preliminary nature of the mechanistic validation, the absence of detailed systemic safety assessments within this model, and the investigation of only a single administration time point, our findings provide compelling preclinical evidence for the therapeutic potential of esketamine. Future research employing more specific molecular tools, assessing dose-response relationships and safety profiles, and optimizing administration timing is warranted to further elucidate the precise mechanisms, define the therapeutic window, and facilitate the potential clinical translation of esketamine for mitigating ALI associated with LIR pathologies.

## Data Availability

All data will be made available by the corresponding author upon reasonable request.

## LIST OF ABBREVIATIONS

ALI: Acute Lung Injury
LIR-ALI: Limb Ischemia-Reperfusion
BALF: Bronchoalveolar Fluid
SPF: Specific Pathogen-Free
SD: Sprague-Dawley
IOD: Integrated Optical Density
